# 3-Benzyl-2-(furan-2-yl)-1,3-thia­zolidin-4-one

**DOI:** 10.1107/S1600536811039432

**Published:** 2011-09-30

**Authors:** Hoong-Kun Fun, Madhukar Hemamalini, Poovan Shanmugavelan, Alagusundaram Ponnuswamy, Rathinavel Jagatheesan

**Affiliations:** a X-ray Crystallography Unit, School of Physics, Universiti Sains Malaysia, 11800 USM, Penang, Malaysia; b Department of Organic Chemistry, School of Chemistry, Madurai Kamaraj University, Madurai 625 021, Tamil Nadu, India; c Department of Chemistry, Thanthai Hans Roever College, Perambalur 621 212, Tamil Nadu, India

## Abstract

In the title compound, C_14_H_13_NO_2_S, the thia­zolidine ring is approximately planar with a maximum deviation of 0.112 (1) Å. The furan ring is disordered over two orientations, with an occupancy ratio of 0.901 (5):0.099 (5). The central thia­zolidine ring makes dihedral angles of 85.43 (8), 87.50 (11) and 87.9 (9)° with the phenyl ring and the major and minor components of the disordered furan ring, respectively. In the crystal, mol­ecules are connected by weak inter­molecular C—H⋯O hydrogen bonds, forming supra­molecular chains parallel to the *b* axis.

## Related literature

For details and applications of thia­zolidine-4-ones, see: Dutta *et al.* (1990 ▶); Jadhav & Ingle (1978 ▶); Gursoy *et al.* (2005 ▶); Rawal *et al.* (2007 ▶); Shrivastava *et al.* (2005 ▶); Look *et al.* (1996 ▶); Anders *et al.* (2001 ▶); Barreca *et al.* (2001 ▶); Diurno *et al.* (1992 ▶). For the stability of the temperature controller used in the data collection, see: Cosier & Glazer (1986 ▶).
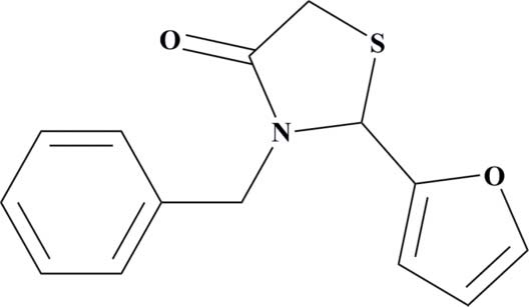


## Experimental

### 

#### Crystal data

C_14_H_13_NO_2_S
*M* *_r_* = 259.31
Monoclinic, 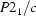

*a* = 13.2901 (2) Å
*b* = 9.6360 (1) Å
*c* = 9.9152 (1) Å
β = 102.855 (1)°
*V* = 1237.95 (3) Å^3^
*Z* = 4
Mo *K*α radiation
μ = 0.25 mm^−1^
*T* = 100 K
0.30 × 0.18 × 0.16 mm

#### Data collection

Bruker SMART APEXII CCD area-detector diffractometer
Absorption correction: multi-scan (*SADABS*; Bruker, 2009 ▶) *T*_min_ = 0.928, *T*_max_ = 0.961
13239 measured reflections
3551 independent reflections
2794 reflections with *I* > 2σ(*I*)
*R*_int_ = 0.028

#### Refinement

*R*[*F* ^2^ > 2σ(*F* ^2^)] = 0.046
*wR*(*F* ^2^) = 0.104
*S* = 1.07
3551 reflections
180 parameters
H-atom parameters constrained
Δρ_max_ = 0.43 e Å^−3^
Δρ_min_ = −0.35 e Å^−3^

### 

Data collection: *APEX2* (Bruker, 2009 ▶); cell refinement: *SAINT* (Bruker, 2009 ▶); data reduction: *SAINT*; program(s) used to solve structure: *SHELXTL* (Sheldrick, 2008 ▶); program(s) used to refine structure: *SHELXTL*; molecular graphics: *SHELXTL*; software used to prepare material for publication: *SHELXTL* and *PLATON* (Spek, 2009 ▶).

## Supplementary Material

Crystal structure: contains datablock(s) global, I. DOI: 10.1107/S1600536811039432/rz2640sup1.cif

Structure factors: contains datablock(s) I. DOI: 10.1107/S1600536811039432/rz2640Isup2.hkl

Supplementary material file. DOI: 10.1107/S1600536811039432/rz2640Isup3.cml

Additional supplementary materials:  crystallographic information; 3D view; checkCIF report

## Figures and Tables

**Table 1:** Hydrogen-bond geometry (Å, °)

*D*—H⋯*A*	*D*—H	H⋯*A*	*D*⋯*A*	*D*—H⋯*A*
C2—H2*A*⋯O2^i^	0.93	2.48	3.355 (3)	158

Symmetry code: (i) 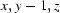.

**Fig. 1.**
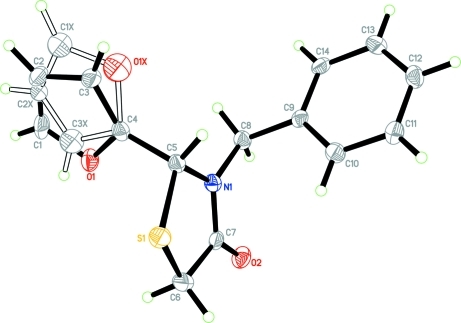
An ORTEP view of the title compound, showing 50% probability displacement ellipsoids. Open bonds indicate the mino component of the disordered furan ring.

**Fig. 2.**
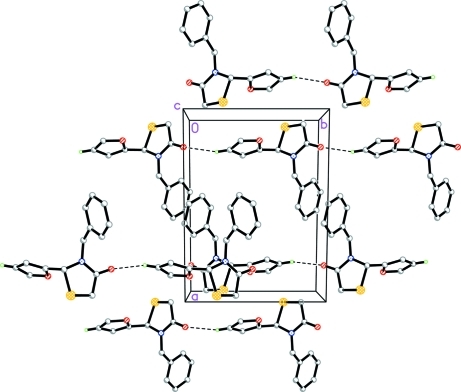
Crystal packing of the title compound viewed along the c axis. Only the major component of the disordered furan ring is shown.

## supplementary crystallographic information

### Comment

One of the main objectives of organic and medicinal chemistry is the design, synthesis and production of molecules having value as human therapeutic agents. During the past decade, combinatorial chemistry has provided access to chemical libraries based on privileged structures with heterocyclic moiety receiving special attention as they belong to a class of compounds with proven utility in medicinal chemistry.  There are numerous biologically active molecules with five membered rings containing two hetero atoms. Among them, thiazolidin-4-ones are the most extensively investigated class of compounds, which have many interesting activity profiles namely bactericidal (Dutta *et al.*, 1990), antifungal (Jadhav & Ingle, 1978), anticonvulsant (Gursoy *et al.*, 2005), anti-HIV (Rawal *et al.*, 2007), antituberculotic (Shrivastava *et al.*, 2005), COX-1 inhibitors (Look *et al.*, 1996), inhibitors of the bacterial enzyme MurB (Anders *et al.*, 2001), non-nucleoside inhibitors of HIV-RT (Barreca *et al.*, 2001) and anti-histaminic agents (Diurno *et al.*, 1992).

The molecular structure of the title compound is shown in Fig. 1.  The thiazolidine (S1/N1/C5–C7) ring is approximately planar, with a maximum deviation of 0.112 (1) Å for atom S1. The furan ring is disordered over two orientations, with an occupancy ratio of 0.901 (5):0.099 (5). The central thiazolidine ring makes dihedral angles of 85.43 (8)°, 87.50 (11) and 87.9 (9)° with the terminal phenyl (C9–C14) ring and the major (O1/C1–C4) and minor (O1X/C1X–C3X/C4) components of the disordered furan ring, respectively. In the crystal structure (Fig. 2),  the molecules are connected by weak intermolecular C—H···O (Table 1) hydrogen bonds forming supramolecular chains parallel to the *b*-axis.

### Experimental

To a well ground intimate mixture of triphenylphosphine (1.1 mmol) and furfuraldehyde, (1.0 mmol) in a microwave vial (10 ml) equipped with a magnetic stirring bar, benzylazide (0.2 g, 1.0 mmol) was added dropwise with stirring. Stirring was continued until liberation of nitrogen ceased and then mercaptoacetic acid, (1.1 mmol), was added to the above mixture and the reaction vessel was sealed with a septum.  It was then placed into the cavity of a focused monomode microwave reactor (CEM Discover, benchmate) and operated at 150°C (temperature monitored by a built-in IR sensor), power 80W for 10 minutes.  The reaction temperature was maintained by modulating the power level of the reactor. The reaction mixture was allowed to stand at room temperature. The residue was then purified by column  chromatography on silica (petrolium/ether-ethylacetate, 94:6 *v*/*v*) to afford 3-benzyl-2-(furan-2-yl)thiazolidin-4-one. Yield: 0.36 g (94%); M.p: 150–151°C. Crystals suitable for X-ray analysis were obtained by slow evaporation of an ethanol solution.

### Refinement

All hydrogen atoms were positioned geometrically [C–H = 0.93–0.98 Å] and were refined using a riding model, with *U*_iso_(H) = 1.2 *U*_eq_(C). The furan ring is disordered over two orientations, with an occupancy ratio of 0.901 (5):0.099 (5).

### Crystal data

**Table:**

C_14_H_13_NO_2_S	*F*(000) = 544
*M**_r_* = 259.31	*D*_x_ = 1.391 Mg m^−^^3^
Monoclinic, *P*2_1_/*c*	Mo *K*α radiation, λ = 0.71073 Å
Hall symbol: -P 2ybc	Cell parameters from 5111 reflections
*a* = 13.2901 (2) Å	θ = 2.6–29.8°
*b* = 9.6360 (1) Å	µ = 0.25 mm^−^^1^
*c* = 9.9152 (1) Å	*T* = 100 K
β = 102.855 (1)°	Block, colourless
*V* = 1237.95 (3) Å^3^	0.30 × 0.18 × 0.16 mm
*Z* = 4	

### Data collection

**Table:**

Bruker SMART APEXII CCD area-detector diffractometer	3551 independent reflections
Radiation source: fine-focus sealed tube	2794 reflections with *I* > 2σ(*I*)
graphite	*R*_int_ = 0.028
φ and ω scans	θ_max_ = 29.9°, θ_min_ = 1.6°
Absorption correction: multi-scan (*SADABS*; Bruker, 2009)	*h* = −18→18
*T*_min_ = 0.928, *T*_max_ = 0.961	*k* = −9→13
13239 measured reflections	*l* = −10→13

### Refinement

**Table:**

Refinement on *F*^2^	Primary atom site location: structure-invariant direct methods
Least-squares matrix: full	Secondary atom site location: difference Fourier map
*R*[*F*^2^ > 2σ(*F*^2^)] = 0.046	Hydrogen site location: inferred from neighbouring sites
*wR*(*F*^2^) = 0.104	H-atom parameters constrained
*S* = 1.07	*w* = 1/[σ^2^(*F*_o_^2^) + (0.0277*P*)^2^ + 1.0975*P*] where *P* = (*F*_o_^2^ + 2*F*_c_^2^)/3
3551 reflections	(Δ/σ)_max_ = 0.001
180 parameters	Δρ_max_ = 0.43 e Å^−^^3^
0 restraints	Δρ_min_ = −0.35 e Å^−^^3^

### Special details

**Table:**

Experimental. The crystal was placed in the cold stream of an Oxford Cryosystems Cobra open-flow nitrogen cryostat (Cosier & Glazer, 1986) operating at 100.0 (1) K.
Geometry. All s.u.'s (except the s.u. in the dihedral angle between two l.s. planes) are estimated using the full covariance matrix. The cell s.u.'s are taken into account individually in the estimation of s.u.'s in distances, angles and torsion angles; correlations between s.u.'s in cell parameters are only used when they are defined by crystal symmetry. An approximate (isotropic) treatment of cell s.u.'s is used for estimating s.u.'s involving l.s. planes.
Refinement. Refinement of F^2^ against ALL reflections. The weighted R-factor wR and goodness of fit S are based on F^2^, conventional R-factors R are based on F, with F set to zero for negative F^2^. The threshold expression of F^2^ > 2σ(F^2^) is used only for calculating R-factors(gt) etc. and is not relevant to the choice of reflections for refinement. R-factors based on F^2^ are statistically about twice as large as those based on F, and R- factors based on ALL data will be even larger.

### Fractional atomic coordinates and isotropic or equivalent isotropic displacement parameters (Å^2^)

**Table:**

	*x*	*y*	*z*	*U*_iso_*/*U*_eq_	Occ. (<1)
S1	0.94899 (3)	0.23126 (5)	0.26095 (5)	0.02134 (12)	
O2	0.82632 (10)	0.47724 (13)	−0.03755 (13)	0.0249 (3)	
N1	0.78175 (10)	0.28671 (15)	0.07263 (15)	0.0176 (3)	
C1	0.85886 (16)	−0.1044 (3)	−0.0489 (2)	0.0257 (6)	0.901 (5)
H1A	0.8838	−0.1380	−0.1231	0.031*	0.901 (5)
C2	0.8142 (2)	−0.1838 (3)	0.0346 (3)	0.0234 (7)	0.901 (5)
H2A	0.8031	−0.2791	0.0288	0.028*	0.901 (5)
C3	0.78759 (17)	−0.0901 (2)	0.1338 (2)	0.0218 (5)	0.901 (5)
H3A	0.7556	−0.1132	0.2051	0.026*	0.901 (5)
O1	0.86228 (11)	0.0324 (2)	−0.00892 (16)	0.0228 (4)	0.901 (5)
C1X	0.7671 (17)	−0.190 (2)	0.096 (2)	0.030 (5)*	0.099 (5)
H1XA	0.7404	−0.2758	0.1125	0.036*	0.099 (5)
C2X	0.828 (2)	−0.160 (3)	0.004 (3)	0.016 (6)*	0.099 (5)
H2XA	0.8446	−0.2253	−0.0560	0.019*	0.099 (5)
C3X	0.8599 (19)	−0.030 (3)	0.009 (3)	0.031 (6)*	0.099 (5)
H3XA	0.9034	0.0087	−0.0426	0.037*	0.099 (5)
O1X	0.7542 (15)	−0.060 (2)	0.1596 (18)	0.038 (5)*	0.099 (5)
C4	0.81768 (12)	0.03740 (19)	0.10405 (18)	0.0188 (3)	
C5	0.81880 (12)	0.17612 (18)	0.17059 (18)	0.0174 (3)	
H5A	0.7748	0.1722	0.2378	0.021*	
C6	0.95721 (13)	0.3595 (2)	0.1314 (2)	0.0241 (4)	
H6A	0.9851	0.4456	0.1751	0.029*	
H6B	1.0020	0.3270	0.0730	0.029*	
C7	0.84910 (12)	0.38244 (18)	0.04616 (17)	0.0177 (3)	
C8	0.67229 (12)	0.29307 (19)	0.00708 (18)	0.0196 (4)	
H8A	0.6477	0.2000	−0.0195	0.024*	
H8B	0.6634	0.3482	−0.0766	0.024*	
C9	0.60718 (12)	0.35483 (17)	0.09945 (17)	0.0164 (3)	
C10	0.64856 (13)	0.44688 (19)	0.20516 (18)	0.0209 (4)	
H10A	0.7185	0.4682	0.2227	0.025*	
C11	0.58667 (13)	0.50767 (19)	0.28530 (18)	0.0218 (4)	
H11A	0.6152	0.5694	0.3555	0.026*	
C12	0.48236 (13)	0.4757 (2)	0.25993 (19)	0.0229 (4)	
H12A	0.4407	0.5156	0.3134	0.027*	
C13	0.44033 (13)	0.3840 (2)	0.1546 (2)	0.0257 (4)	
H13A	0.3704	0.3625	0.1374	0.031*	
C14	0.50243 (13)	0.32405 (19)	0.07495 (19)	0.0213 (4)	
H14A	0.4737	0.2627	0.0045	0.026*	

### Atomic displacement parameters (Å^2^)

**Table:**

	*U*^11^	*U*^22^	*U*^33^	*U*^12^	*U*^13^	*U*^23^
S1	0.01828 (19)	0.0221 (2)	0.0216 (2)	0.00020 (16)	0.00008 (15)	−0.00073 (18)
O2	0.0310 (7)	0.0178 (7)	0.0270 (7)	0.0029 (5)	0.0089 (5)	0.0037 (5)
N1	0.0153 (6)	0.0165 (7)	0.0208 (7)	0.0019 (5)	0.0037 (5)	0.0018 (6)
C1	0.0270 (10)	0.0223 (12)	0.0268 (11)	0.0083 (9)	0.0033 (9)	−0.0086 (10)
C2	0.0199 (12)	0.0136 (13)	0.0337 (17)	−0.0003 (9)	−0.0006 (11)	0.0005 (11)
C3	0.0173 (9)	0.0179 (11)	0.0290 (11)	−0.0006 (8)	0.0021 (8)	0.0011 (9)
O1	0.0266 (8)	0.0204 (10)	0.0232 (8)	0.0048 (7)	0.0093 (6)	−0.0014 (7)
C4	0.0153 (7)	0.0204 (9)	0.0202 (8)	0.0026 (6)	0.0027 (6)	0.0002 (7)
C5	0.0155 (7)	0.0170 (8)	0.0203 (8)	0.0019 (6)	0.0051 (6)	0.0021 (7)
C6	0.0187 (8)	0.0208 (10)	0.0330 (10)	−0.0017 (7)	0.0061 (7)	0.0024 (8)
C7	0.0206 (7)	0.0131 (8)	0.0204 (8)	0.0020 (6)	0.0069 (6)	−0.0021 (7)
C8	0.0159 (7)	0.0207 (9)	0.0211 (8)	0.0018 (6)	0.0018 (6)	−0.0019 (7)
C9	0.0176 (7)	0.0118 (8)	0.0189 (8)	0.0022 (6)	0.0020 (6)	0.0031 (6)
C10	0.0160 (7)	0.0223 (9)	0.0230 (9)	0.0014 (6)	0.0011 (6)	−0.0005 (7)
C11	0.0238 (8)	0.0201 (9)	0.0206 (8)	0.0029 (7)	0.0026 (7)	−0.0028 (7)
C12	0.0232 (8)	0.0204 (9)	0.0266 (9)	0.0070 (7)	0.0089 (7)	0.0035 (7)
C13	0.0174 (8)	0.0254 (10)	0.0355 (10)	−0.0007 (7)	0.0081 (7)	0.0001 (8)
C14	0.0200 (8)	0.0151 (9)	0.0277 (9)	−0.0039 (6)	0.0032 (7)	−0.0031 (7)

### Geometric parameters (Å, °)

**Table:**

S1—C6	1.8032 (19)	O1X—C4	1.453 (18)
S1—C5	1.8410 (16)	C4—C5	1.489 (2)
O2—C7	1.226 (2)	C5—H5A	0.9800
N1—C7	1.351 (2)	C6—C7	1.512 (2)
N1—C5	1.452 (2)	C6—H6A	0.9700
N1—C8	1.457 (2)	C6—H6B	0.9700
C1—C2	1.357 (4)	C8—C9	1.515 (2)
C1—O1	1.375 (3)	C8—H8A	0.9700
C1—H1A	0.9300	C8—H8B	0.9700
C2—C3	1.436 (4)	C9—C10	1.390 (2)
C2—H2A	0.9300	C9—C14	1.391 (2)
C3—C4	1.344 (3)	C10—C11	1.394 (2)
C3—H3A	0.9300	C10—H10A	0.9300
O1—C4	1.380 (2)	C11—C12	1.387 (2)
C1X—C2X	1.37 (3)	C11—H11A	0.9300
C1X—O1X	1.43 (3)	C12—C13	1.387 (3)
C1X—H1XA	0.9300	C12—H12A	0.9300
C2X—C3X	1.32 (4)	C13—C14	1.389 (3)
C2X—H2XA	0.9300	C13—H13A	0.9300
C3X—C4	1.36 (3)	C14—H14A	0.9300
C3X—H3XA	0.9300		
			
C6—S1—C5	92.90 (8)	C4—C5—H5A	108.6
C7—N1—C5	119.36 (13)	S1—C5—H5A	108.6
C7—N1—C8	121.58 (15)	C7—C6—S1	107.31 (12)
C5—N1—C8	119.06 (14)	C7—C6—H6A	110.3
C2—C1—O1	110.8 (2)	S1—C6—H6A	110.3
C2—C1—H1A	124.6	C7—C6—H6B	110.3
O1—C1—H1A	124.6	S1—C6—H6B	110.3
C1—C2—C3	105.7 (2)	H6A—C6—H6B	108.5
C1—C2—H2A	127.1	O2—C7—N1	124.45 (15)
C3—C2—H2A	127.1	O2—C7—C6	123.23 (16)
C4—C3—C2	107.1 (2)	N1—C7—C6	112.32 (15)
C4—C3—H3A	126.5	N1—C8—C9	113.27 (14)
C2—C3—H3A	126.5	N1—C8—H8A	108.9
C1—O1—C4	105.93 (18)	C9—C8—H8A	108.9
C2X—C1X—O1X	105 (2)	N1—C8—H8B	108.9
C2X—C1X—H1XA	127.6	C9—C8—H8B	108.9
O1X—C1X—H1XA	127.6	H8A—C8—H8B	107.7
C3X—C2X—C1X	114 (3)	C10—C9—C14	118.73 (16)
C3X—C2X—H2XA	122.9	C10—C9—C8	121.56 (15)
C1X—C2X—H2XA	122.9	C14—C9—C8	119.66 (15)
C2X—C3X—C4	107 (2)	C9—C10—C11	120.90 (16)
C2X—C3X—H3XA	126.4	C9—C10—H10A	119.5
C4—C3X—H3XA	126.4	C11—C10—H10A	119.5
C1X—O1X—C4	105.1 (15)	C12—C11—C10	119.73 (17)
C3—C4—C3X	84.6 (13)	C12—C11—H11A	120.1
C3—C4—O1	110.41 (18)	C10—C11—H11A	120.1
C3X—C4—O1X	108.5 (14)	C13—C12—C11	119.79 (16)
O1—C4—O1X	132.6 (8)	C13—C12—H12A	120.1
C3—C4—C5	134.18 (18)	C11—C12—H12A	120.1
C3X—C4—C5	140.1 (13)	C12—C13—C14	120.16 (16)
O1—C4—C5	115.28 (16)	C12—C13—H13A	119.9
O1X—C4—C5	111.3 (8)	C14—C13—H13A	119.9
N1—C5—C4	113.19 (14)	C13—C14—C9	120.68 (17)
N1—C5—S1	104.82 (11)	C13—C14—H14A	119.7
C4—C5—S1	112.97 (11)	C9—C14—H14A	119.7
N1—C5—H5A	108.6		
			
O1—C1—C2—C3	0.1 (2)	O1—C4—C5—N1	49.22 (19)
C1—C2—C3—C4	−0.1 (2)	O1X—C4—C5—N1	−121.5 (8)
C2—C1—O1—C4	0.0 (2)	C3—C4—C5—S1	105.7 (2)
O1X—C1X—C2X—C3X	4(3)	C3X—C4—C5—S1	−57.9 (17)
C1X—C2X—C3X—C4	−2(3)	O1—C4—C5—S1	−69.72 (17)
C2X—C1X—O1X—C4	−4(2)	O1X—C4—C5—S1	119.6 (8)
C2—C3—C4—C3X	−6.0 (11)	C6—S1—C5—N1	−16.21 (12)
C2—C3—C4—O1	0.1 (2)	C6—S1—C5—C4	107.48 (13)
C2—C3—C4—O1X	153.3 (18)	C5—S1—C6—C7	15.65 (13)
C2—C3—C4—C5	−175.46 (19)	C5—N1—C7—O2	177.75 (16)
C2X—C3X—C4—C3	8.7 (19)	C8—N1—C7—O2	−3.4 (3)
C2X—C3X—C4—O1	−159 (4)	C5—N1—C7—C6	−2.0 (2)
C2X—C3X—C4—O1X	−1(2)	C8—N1—C7—C6	176.90 (15)
C2X—C3X—C4—C5	177.0 (13)	S1—C6—C7—O2	169.34 (14)
C1—O1—C4—C3	0.0 (2)	S1—C6—C7—N1	−10.94 (18)
C1—O1—C4—C3X	14 (2)	C7—N1—C8—C9	−99.90 (19)
C1—O1—C4—O1X	−15.4 (10)	C5—N1—C8—C9	78.97 (19)
C1—O1—C4—C5	176.43 (14)	N1—C8—C9—C10	25.8 (2)
C1X—O1X—C4—C3	−18.8 (11)	N1—C8—C9—C14	−157.01 (16)
C1X—O1X—C4—C3X	3.0 (18)	C14—C9—C10—C11	−0.1 (3)
C1X—O1X—C4—O1	16.1 (18)	C8—C9—C10—C11	177.10 (16)
C1X—O1X—C4—C5	−175.3 (11)	C9—C10—C11—C12	0.3 (3)
C7—N1—C5—C4	−110.10 (17)	C10—C11—C12—C13	−0.3 (3)
C8—N1—C5—C4	70.99 (19)	C11—C12—C13—C14	0.1 (3)
C7—N1—C5—S1	13.44 (18)	C12—C13—C14—C9	0.1 (3)
C8—N1—C5—S1	−165.46 (12)	C10—C9—C14—C13	−0.1 (3)
C3—C4—C5—N1	−135.4 (2)	C8—C9—C14—C13	−177.32 (17)
C3X—C4—C5—N1	61.0 (17)		

### Hydrogen-bond geometry (Å, °)

**Table:**

*D*—H···*A*	*D*—H	H···*A*	*D*···*A*	*D*—H···*A*
C2—H2A···O2^i^	0.93	2.48	3.355 (3)	158

Symmetry codes: (i) *x*, *y*−1, *z*.
